# When is it good to feel bad? How sadness and fear differ in their effects on routine development

**DOI:** 10.3389/fpsyg.2023.1141454

**Published:** 2023-11-30

**Authors:** Jutta Stumpf-Wollersheim, Patrick J. Oehler, Marlen Rimbeck, Matthias Spörrle, Isabell M. Welpe

**Affiliations:** ^1^Chair of International Management and Corporate Strategy, Technische Universität Bergakademie Freiberg, Freiberg, Germany; ^2^Chair for Strategy and Organization, Technical University of Munich, Munich, Germany; ^3^Private University Schloss Seeburg, Seekirchen am Wallerseee, Austria

**Keywords:** affect, organizational routines, decision-making, organizational evolution and change, group processes and performance, cognitive stimuli

## Abstract

**Introduction:**

This study follows recent calls to explore the emotional foundations of routine development. Routine development forms a nexus between stability and change and is thus crucial for studying organizational decision-making and organizational change. Individuals and teams going through organizational change often experience sadness and fear.

**Methods:**

We conducted a laboratory experiment with 84 teams to study the effect of sadness and fear on routine development.

**Results and discussion:**

In the sadness condition, we observed positive effects on repetitiveness, speed, reliability, and attentiveness in action. Teams experiencing fear reacted better to ‘performance traps’ in which pre-established routines are ineffective. Our findings show how the behaviors elicited by sadness and fear might ultimately affect team behavior, and therefore managerial practices.

## 1 Introduction

In order to understand organizational decision-making, the exploration of routine development is central. In the context of the Carnegie perspective, routines represent the basic unit for analyzing decision behavior (Gavetti et al., 2007). Routines are examined as “repetitive, recognizable patterns of interdependent actions, carried out by multiple actors” (Feldman and Pentland, 2003, p. 95). Routine development provides stability through repetitiveness and allows for quick and reliable performances (Cohen and Bacdayan, 1994) as well as effective cooperation in teams (Annosi et al., 2020; Blume et al., 2021). Understanding routine development is crucial to understanding whether and how organizations and teams take decisions under organizational change, as routines may simultaneously represent inhibitors as well as sources of change (Feldman and Pentland, 2003).

Both early research on human behavior (Dewey, 1922) and routine research provide theoretical indications that emotions are connected with the development of behavioral patterns and routines (Adler and Obstfeld, 2007; Krisberga-Sinigoi et al., 2019; Zietsma et al., 2019). Further, in the context of the Carnegie perspective, it is also suggested to study emotional behavior in order to better understand organizational decision-making (Gavetti et al., 2007). One of the few studies that address routines related to emotions stems from Døjbak Håkonsson et al. (2016), finding that negative emotions generally relate to a lower likelihood of adaption to new routines than positive emotions and may thus inhibit organizations from changing their routines. However, while Døjbak Håkonsson et al.’s (2016) study is highly valuable, as it suggests that the evolution of routines is in part shaped by highly contagious negative emotions (Bartel and Saavedra, 2000; Barsade, 2002), our understanding of the differential and microfoundational effects of distinct negative emotions on routine development remains limited. Understanding these effects is important for developing “new theory and research […] to shed light on the generative mechanisms through which firms might […] harness the […] emotional capacities of individuals and groups” (Hodgkinson and Healey, 2014, p. 1306).

The two distinct negative emotions sadness and fear are particularly relevant in the context of routine development. First, sadness and fear are likely to result in different effects on routine development. For instance, lab studies show that whereas sadness relates to uncertainty acceptance, fear relates to uncertainty avoidance (Raghunathan and Pham, 1999; Lerner and Keltner, 2001). Second, sadness and fear are particularly likely to be experienced in the context of routine development. Routine development is closely connected to organizational decision-making under change conditions, which is often accompanied by sadness and fear (Kabanoff et al., 1995; Fugate et al., 2002). While developing routines during times of change, organizational members are likely to feel sad about leaving a past state, for instance, due to layoffs of beloved colleagues or due to the breakup of their team (Appelbaum et al., 2000; Basch and Fisher, 2000), and they often experience fear about the future, for instance, fear of losing their jobs or situational control (Appelbaum et al., 2000). Third and finally, sadness and fear are among the most often observed forms of emotional distress (Selye, 1956; Raghunathan and Pham, 1999) and may, for instance, be caused by dysfunctional supervision in change contexts (Oh and Farh, 2017).

Despite the relevance of sadness and fear in contexts in which routine development occurs, so far we do not know how they affect routine development. This research gap is regrettable given the potential consequences of sadness and fear for routine development. In the context of the Carnegie perspective, we follow repeated calls in the extant literature to “also account for emotions […] to complete the microfoundations of our theories” (Hodgkinson and Healey, 2011). At the same time, we follow Barsade and Gibson’s (2007, p. 52) call, who note that “[r]esearch and practice should be directed to the important questions of, “Under what conditions can negative affective responses lead to positive organizational outcomes?.” Specifically, we ask, how do sadness and fear differentially affect routine development? We use a laboratory experiment to causally address this research question. Our findings lead to a better understanding of the mechanisms through which negative emotions affect routine development, thus simultaneously increasing the comprehension of organizational decision-making under change. Our experimental study provides causal evidence that the individual dimensions of routine development processes are differently affected by sadness and fear and thus suggest that distinct emotions as well as different dimensions of routine development should be differentiated in order to understand the effects of negative emotions on routine development. Our findings may lead to the development of more emotion-sensitive change practices and might sensitize organizations to better understand and predict the effects of negative emotions in change processes.

## 2 Theoretical foundation and relevance for the Carnegie perspective

### 2.1 Routine development and its operationalization

Well-known representatives of the Carnegie perspective argue that rational decision-making at the organizational and individual level is limited by various factors. These factors include, for example, that knowledge about specific circumstances and consequences is never complete and not all behavioral alternatives can be fully addressed (Simon, 1947). Simon’s insights highlight the fundamental role of bounded rationality in shaping decision-making within organizations, including that (negative) emotions influence the rationality of decisions. Considering the behavioral theory of the firm according to Cyert and March (1963), routines are required “to deal with the cognitive constraints posed by bounded rationality” (Pentland and Hærem, 2015, p. 475). In other words, decision-making by managers and employees is defined by rule-driven behavior (De Boer and Zandberg, 2012), which is reflected in routine development. Simultaneously, individual behavior and subsequent decision-making are influenced by individually perceived stimuli (March and Simon, 1958; Tosi, 2008). These stimuli encompass, for instance, changes in the external or internal firm environment, which may evoke different perceptions, expectations, and emotions, and consequently unintended behavioral responses. Thus, it is highly relevant to examine routine development, as a central unit of analysis for exploring heuristic decision-making under the influence of (negative) stimuli induced by organizational changes.

The literature on routine development differentiates between the emergence of routines and the adaptation of existing routines. Accordingly, the behavioral theory describes two processes that address both aspects of routine development. The first process refers to the emergence of operating routines and of ecologies of operating routines as repetitive practices that evolve through internal dynamics (Parmigiani and Howard-Grenville, 2011). The second process describes the external modification of operating routines through dynamic capabilities, i.e., “a learned and stable pattern of collective activity through which the organization systematically generates and modifies its operating routines in pursuit of improved effectiveness” (Zollo and Winter, 2002: 340). Both the emergence and the adaptation of existing routines are closely intertwined, and to understand routine development, both processes need to be analyzed jointly (Levinthal and Rerup, 2006).

To determine an operationalization of routine development, one might draw from microfoundational perspectives on routines (Felin et al., 2012, p. 1352). Microfoundational studies of routines have found helpful means to operationalize operating routines, their emergence, and the mechanisms through which they are regulated. For instance, in their pioneering experimental work on organizational routines, Cohen and Bacdayan (1994) introduced four dimensions to operationalize routines and their development. These four dimensions show overlaps with alternative operationalizations of routines (Pentland, 2003a,b; Becker, 2005; Laureiro-Martinez, 2014) and have been used to operationalize both operating routines (Cohen and Bacdayan, 1994) and dynamic capabilities (Wollersheim and Heimeriks, 2016). Considering studies on routine development at the individual level (e.g., Laureiro-Martinez, 2014) and recognizing that people are complex beings, it becomes clear that introducing (negative) emotions can have distinct effects on their routinization. Three out of the four dimensions introduced by Cohen and Bacdayan (1994) capture the emergence of operating routines by means of three important characteristics of routines: (1) repetitiveness in action, (2) speed in action, and (3) reliability in action. The fourth of Cohen and Bacdayan’s (1994) dimensions captures to what extent teams are able to recognize ‘performance traps’ and, accordingly, to attentively modify their routines in situations in which adjustments may lead to increased performance. This fourth dimension, (4) attentiveness in action, provides a meaningful operationalization of routine modification.^1^ All four dimensions capture different facets of routine development, and (as we discuss below) they may be differently affected by sadness and fear.

### 2.2 The effects of sadness and fear on routine development

#### 2.2.1 Repetitiveness in action

Routine development involves the emergence of action sequences, which through repetition develop into operating routines and which, due to their repetitiveness, are recognizable as such (Feldman and Pentland, 2003; Becker, 2004). In the context of our research model, repetitiveness in action corresponds to the question: Which operating routines or ecologies of operating routines develop, and how much control do they provide?

There is some indication that sadness and fear may affect repetitiveness in action. Emotions generally “provide[…] the motivating force driving strong commitment to novel choices” and actions (Hodgkinson and Healey, 2014: 1310), while negative emotions may decrease the likelihood of teams adopting novel actions (Døjbak Håkonsson et al., 2016). Consequently, both sadness and fear are likely to result in the development of more-repetitive operating routines. This expectation is supported by appraisal perspectives on emotions, which associate both sadness and fear with high levels of situational control (Smith and Ellsworth, 1985). That is, in change processes, sad and fearful teams and their members are likely to attribute the control of their situation to uncontrollable circumstances (Smith and Ellsworth, 1985), for instance, to the market environment or to the management. We may expect that teams of sad and fearful individuals restore a feeling of control by increasing the repetitiveness of their actions (Nelson and Winter, 1982; Becker, 2004). Consistent with this prediction, Staw et al.’s (1981) threat-rigidity thesis suggests that external threats, which tend to be accompanied by fear, generally lead to more repetitiveness in behavior. Further, Gill and Burrow (2018) reveal that fear leads to conforming behavior and accurate reproduction of familiar working practices, suggesting fear to be an influencing factor for organizational performance. In conclusion, we expect the development of more repetitive, recognizable, and thus controllable operating routines for teams whose members share a feeling of sadness or fear relative to teams whose members do not feel these emotions. However, we expect no differences between sadness and fear regarding repetitiveness in action.

#### 2.2.2 Speed in action

Routine development allows “for the rapid processing of large amounts of information with little effort” (Laureiro-Martinez, 2014, p. 1113) and for economizing on cognitive resources (Becker, 2004). While developing routines, the actors store the components of the operating routines in their procedural memory (Cohen and Bacdayan, 1994). This ‘off-loading’ enables them to act at increasingly higher speeds and to increase their output per unit of time (Cohen and Bacdayan, 1994; Healey et al., 2015). Hence, routine development can be associated with increases in the speed in action (Cohen and Bacdayan, 1994). Overall, speed in action corresponds to the question: How automatically are operating routines executed, i.e., how developed is the execution of operating routines?

There is some indication that sadness and fear may affect speed in action. For instance, sadness has been associated with local impatience, i.e., sad individuals tend to seek instant gratification when facing choices between immediate and future payoffs, an observation that Lerner et al. (2013) denote as ‘myopic misery’. In a change context, sadness may thus translate into an increased tendency to develop operating routines–quick and reliable behavioral patterns that may provide instant gratification (Cohen and Bacdayan, 1994). Likewise, with regard to fear, Vuori and Huy (2016) find that structurally based fear within organizations (e.g., about the future of the company) may lead to temporal myopia, i.e., a focus on short-term activities and failure at implementing long-term activities. In their case study, fear, i.e., the “dread of impending disaster and an intense urge to defend oneself, primarily by getting out of the situation” (Öhman, 2008, p. 710), pressured organizational members to act urgently (Lazarus, 1991; Vuori and Huy, 2016). Thus, with both sadness and fear, we may expect increases in the speed at which operating routines are enacted. In the extant literature, we have found no indication of differences between sadness and fear regarding their effects on speed in action.

#### 2.2.3 Reliability in action

Routine development is targeted toward reliability in action, i.e., toward reducing any risk and uncertainty attached to organizational actions (Becker, 2004). Operating routines tend to be highly reliable, and their outcomes are almost certain (Cyert and March, 1963; Cohen and Bacdayan, 1994). Accordingly, routine development reduces the emotional costs that result from risk and uncertainty (Cohen and Bacdayan, 1994; Laureiro-Martínez et al., 2015). In fact, it has been argued that routine development may be “viewed as an uncertainty decreasing strategy” (Becker, 2004, p. 658). Reliability in action reveals how well-developed a routine is at fulfilling this function, and thus, it corresponds overall to the question: How functionally developed are operating routines?

There is an indication that sadness and fear may affect demands for reliability in action (Delgado-García et al., 2010). For instance, sadness generally relates to more uncertainty acceptance and to more risk taking and, accordingly, to a comparatively decreased demand for reliable actions that reduce uncertainty and risk (Raghunathan and Pham, 1999). In contrast, fear relates to uncertainty avoidance and to less risk taking and, accordingly, to a comparatively increased demand for reliability (Raghunathan and Pham, 1999; Lerner and Keltner, 2001; Liu and Perrewe, 2005). In a change context, we may thus expect a lower demand for reliability and hence a lower tendency toward the development of reliable operating routines when sadness is experienced and a higher demand for reliability and hence a higher tendency toward the development of reliable operating routines when fear is experienced.

#### 2.2.4 Attentiveness in action

Routine development draws from collective activities–dynamic capabilities (Levinthal and Rerup, 2006)–that are dedicated to the creation and modification of operating routines (Zollo and Winter, 2002). Whereas dynamic capabilities themselves may represent mindless activities that are unknown to their actors, they shape operating routines through mindfulness and deliberation in action (Zollo and Winter, 2002) by disciplining collective attention toward operating routines and their enactments (Weick and Sutcliffe, 2006). From a cognitive perspective, the dynamic capability concept is matched by the concept of attention control (Laureiro-Martínez et al., 2015). Dynamic capabilities draw from individuals’ ability to focus their attention on activities that improve effectiveness. For instance, previous research has shown that individual attention guides choices between exploration and exploitation (Laureiro-Martínez et al., 2015). Likewise, dynamic capabilities direct collective attention toward the creation and modification of operating routines (Weick and Sutcliffe, 2006). They allow one to find the optimal balance between stability and change, i.e., to understand when operating routines do not require attention and when they should be attentively enacted and modified. Dynamic capabilities become visible through the attentiveness that is put at work in the enactment and modification of operating routines in situations where routines require attention. Overall, attentiveness in action corresponds to the question: How effectively are operating routines enacted and modified to match the dynamics of their environment?

There is some indication that sadness and fear may affect attentiveness in action. In general, Baldessarelli et al. (2022) states that emotions affect the emergence and willingness to maintain routines. Negative emotions may lead to a modification of routines, especially in the way how patterns of action are realized. In this context, Soderstrom and Weber (2020) dealt with the influence of emotions on integration processes and revealed that the experience of an emotional alignment may affect longer-term motivation as well as commitment structures. These findings can be transferred to our study context in that negatively experienced emotions, such as fear and sadness, can have a lasting and potentially harmful effect on subsequent interactions and routine development. More specifically, Smith and Ellsworth (1985) generally associate sadness with comparatively lower levels of attention and fear with comparatively higher levels of attention. Gable and Harmon-Jones (2010) find that emotions associated with low motivational intensity (sadness) lead to widened attention, whereas emotions associated with high motivational intensity (fear) lead to narrowed attention. This finding implies that sadness might shift the focus of attention away from local stimuli toward global stimuli–for instance, away from the regulation of operating routines to the environment (e.g., toward issues not related to the task at hand). In contrast, fear is likely to lead to an attention shift from the environment toward the regulation of operating routines.

### 2.3 Our research model

**Figure 1:**
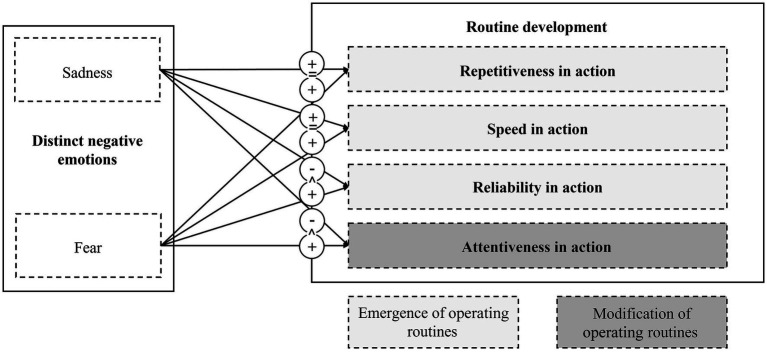
Expected effects of sadness and fear on different dimensions of routine development. The figure illustrates the expected effects of the distinct emotions sadness and fear on different dimensions of routine development. = denotes no effect; + denotes positive effect; − denotes negative effect; > denotes more positive effect when compared to other emotion rather than to control condition.

Building on previous studies of routines, our research model features three different dimensions that capture the emergence of operating routines: (1) repetitiveness in action, (2) speed in action, and (3) reliability in action (Cohen and Bacdayan, 1994). Moreover, we operationalize the regulation of operating routines as a team’s capability to (4) attentively modify operating routines in order to optimize performance. All four dimensions of routine development may be subject to emotional influences, and we expect several differences in the effects of sadness and fear. Figure 1 presents our research model.

## 3 Data and methods

### 3.1 Task

As an experimental task, we used the Target the Two (TTT) card game developed by Cohen and Bacdayan (1994), which has already been used in several other studies to exemplify organizational routines (Egidi and Narduzzo, 1997; Wollersheim and Heimeriks, 2016; Oehler et al., 2019). TTT shares essential aspects with typical routine development situations in organizational settings (e.g., asymmetry of authorities, information asymmetry) and thus serves as a well-recognized laboratory-suited analog of organizational routine development. The game features two team members who are randomly assigned to each other and who need to quickly develop a new routine at solving repeated tasks, which vary slightly over time. Specifically, the card game involves six cards in total (2♥, 3♥, 4♥ and 2♣, 3♣, 4♣). Four of these cards lie on the playing board, and the other two cards are assigned as personal cards to each of the two team members. That is, each team member holds one personal card, which cannot be seen by the other team member. The remaining cards are on the playing board, with two lying face-up and two lying face-down. One of the face-up cards occupies a special position, the target position. The team members’ common goal is to put 2♥ in the target position as quickly as possible and with the least possible number of moves. They alternately exchange their personal card with one of the cards on the playing board until the relative hand is completed–i.e., until 2♥ is placed in the target position. This process requires coordination, given that a special rule applies to the target position. The special rule differs depending on the authority the respective team member represents in the card game: one of the team members is given the authority of a Numberkeeper, which means that he or she can only exchange his or her personal card with the card in the target position if the cards are of the same number; the other team member is given the authority of a Colorkeeper, which means that he or she can only exchange his or her personal card with the card in the target position if his personal card has the same suit as the one in the target position. In each hand, the Colorkeeper moves first. In total, TTT involves 40 hands with various card constellations, and takes up to 40 min. Following Cohen and Bacdayan (1994), we instructed the teams to play up to 40 hands of TTT while not exceeding the maximum time frame of 40 min. Twenty-seven of the 40 original card constellations conceived by Cohen and Bacdayan (1994) are designed in a way that allows both Numberkeepers and Colorkeepers to effectively target the 2♥ card in the target field. In these hands, teams need to coordinate on one authority (Numberkeeper or Colorkeeper), who places the 2♥ card in the target field, whereas the other team member assumes a supportive role (Egidi, 1996; Egidi and Narduzzo, 1997). Teams can only succeed in these tasks if they find a way to coordinate their respective authorities. Because they are urged not to communicate openly, team members may implicitly communicate through ‘signal cards’ to inform the team member about intended actions (Egidi, 1996; Egidi and Narduzzo, 1997).

### 3.2 Procedure

#### 3.2.1 Introduction

On their arrival in the experimental laboratory, participants were assigned a computer. We then introduced our participants to the general background, procedure, and incentive structure of the experiment. The following computerized training included a written explanation of the rules of the game and a sample hand, which illustrated the rules of the game. The computerized training was followed by a short question-and-answer session. In addition to answering the questions raised publicly, the experimenter repeated answers to some general questions that–according to pretests and observations from other studies with this card game (Wollersheim and Heimeriks, 2016)–appeared regularly. After the question-and-answer session, we distributed printed rule cards indicating the respective roles (i.e., Colorkeeper or Numberkeeper) and summarizing the rule that applied to the respective role of the participants. The participants were randomly allocated to teams, which–without their knowledge–were assigned to our three different emotion induction conditions.

#### 3.2.2 Emotional manipulation

We implemented the emotion intervention by inducing fear, sadness, or no specific emotions. We only induced one emotion per team (i.e., emotions were not mixed within teams). After introducing the experimental procedure, we instructed the participants to write a short essay for 5 min. Specifically, we instructed them to write a detailed description of an event that made them feel either deeply sad (sadness condition) or afraid (fear condition) or one that regularly occurs and does not have any obvious emotional influence (control condition). This emotion induction procedure is widely used in economic studies (e.g., Nelissen et al., 2011; Siedlecka and Denson, 2019) in which decisions have to be made directly after finishing the writing task. Because this study needed to sustain these emotions for up to 40 min, we additionally used a combination of music and pictures for the emotion induction during the game (Lench et al., 2011). Previous research has shown that combining music and pictures is more effective for inducing emotions than using pictures alone (Baumgartner et al., 2006b), and several studies have successfully combined auditory and visual stimuli for inducing emotions (Drace et al., 2009; Haase and Silbereisen, 2011). To avoid that participants notice the emotional manipulation and to ensure that the results of the experiment are influenced in this respect, the emotional state was not queried until the end of the experiment.^2^

During the experimental task, we played music that was used in previous research (Krumhansl, 1997; Etzel et al., 2006) to the participants via headphones (headphones were also used in previous studies, e.g., Stephens et al., 2010). Specifically, the musical stimulus material consisted of soundtrack (Etzel et al., 2006) and classical music (Mayer et al., 1995; Baumgartner et al., 2006a) to induce sadness and fear. The music excerpts were played in a random order. Participants in the control condition wore headphones without listening to music (Niedenthal et al., 2001), because “neutral music does not exist” (Baumgartner et al., 2006a, p. 41).

The visual stimulus material consisted of 10 pictures per experimental condition (i.e., 10 pictures for inducing sadness, 10 pictures for inducing fear, and 10 pictures for the control condition). Most of the pictures were taken from the International Affective Picture System (IAPS). Those pictures that were not taken from the IAPS were collected from the internet (IAPS pictures were also supplemented with pictures that had been collected by the authors in Baumgartner et al., 2006a). During the experimental task, the pictures regularly popped up on participants’ computer screens. Participants in the control condition were exposed to neutral objects. All pictures that were used for the main study had been pretested. For the visual induction, each picture was presented at the center of the screen for 30 ms (e.g., Soussignan et al., 2010), and there was a time lag of 10 s between the picture presentations. The pictures were presented in random order.

Because this study induced emotions for such a long time frame, we pretested the whole emotion induction procedure. Participants (*N* = 72) who did not take part in the main study were asked after five minutes, after 20 min, and after completion of the game to what degree they currently felt sad and to what degree they currently felt afraid. As shown in Table 1, the manipulation was successful across all time spans, indicating that the combination of different stimuli allowed the emotions to be maintained over the entire duration of the experiment. In the main study, we conducted further manipulation checks, which were all successful. Yet, in contrast to the pre-tests, we tested emotions only at the end of the study to avoid distractions from the task. Accordingly, the manipulation checks could not have influenced routine development in our main sessions.

**Table 1:** Manipulation check pre-test.

	Control condition		Sadness condition			Control condition		Fear condition	
	Mean	SD	Mean	SD		Mean	SD	Mean	SD
*Reported sadness*					*Reported fear*				
*After 5 min.*	1.21	1.62	4.08	2.43	*After 5 min.*	1.63	2.26	3.50	3.31
*After 20 min.*	1.46	1.56	3.71	2.68	*After 20 min.*	1.04	1.46	2.36	2.63
*After the game*	1.25	1.57	3.21	2.60	*After the game*	0.54	0.78	1.54	2.06

#### 3.2.3 Measurement of routine development

Following Wollersheim and Heimeriks (2016) we used a computerized version of TTT (Cohen and Bacdayan, 1994). Our version of TTT was programmed as a client–server-solution that displayed cards in the same order on each screen as the original game. We measured routine development in the game by means of four different dependent variables, of which the first three captured the emergence of operating routines by means of (1) repetitiveness in action, (2) speed in action, and (3) reliability in action, and of which the fourth captured the modification of operating routines by means of (4) attentiveness in action.

(1) To measure repetitiveness in action, we identified distinct action patterns and their repetitions in the TTT game (Cohen and Bacdayan, 1994). In the game, action patterns can be identified and differentiated according to the field positions with which team members exchange the cards in their hands in their efforts to solve TTT. Every move in TTT either represents a card exchange with a field position on the virtual table or an activation of the pass button. Individual moves may, hence, be aggregated into action sequences that capture the chronological order of moves over the course of one hand. We can use these orders to differentiate distinct action sequences and their repeated enactment throughout the game. Specifically, we analyzed either the last four moves of a hand if a hand was solved within four or more moves or the last three moves of a hand if the hand was solved within three moves (Cohen and Bacdayan, 1994). We chose this approach because the first few moves of each action string tend to be very specific to the different constellations of cards on the playing field, whereas the last few moves of each hand can be replicated throughout various constellations of cards. Thus, for each team and each hand, we determined the combination of the last three to four moves that led to the solution of the hand. The respective solutions are stored in our variable ‘distinct action sequences’. To determine repetitiveness in these distinct action sequences, we counted the recurrences of each ‘distinct action sequence’ for each team by means of our variable ‘repetitiveness of distinct action sequences’.

(2) To measure speed in action, we followed Cohen and Bacdayan (1994) in measuring the ‘average move time per hand’ and changes in this variable throughout the TTT game (Laureiro-Martinez, 2014). That is, for each hand played by each team, we individually assessed the average time it took the team members to execute the moves of this hand. Measuring speed in action for each hand separately enabled us to assess how speed in action changed over the course of the TTT game.

(3) To measure reliability in action, we analyzed the ‘deviation in number of moves relative to the best team’. That is, for each team and each hand, we determined the difference in the number of moves required by the analyzed team and the number of moves required by the team that required the lowest number of moves for the respective hand. Thereby, we refine Cohen and Bacdayan’s (1994) measure for reliability, which is limited in its explanatory power, in that it basically only compares two out of all participating teams to each other.

(4) To measure attentiveness in action, we looked at occasional suboptimality. Cohen and Bacdayan (1994) find that the development of operating routines, such as the so-called UU*T sequence (named after the sequence of activated fields *U*p, *U*p, *Anything, *T*arget), may contribute to occasional suboptimality. In their experimental setting, players tend to stick to pre-established action patterns even in situations in which different solutions would have been more efficient. Yet, Wollersheim and Heimeriks (2016) find that teams playing TTT may benefit from dynamic capabilities that are reflected in an increased attentiveness in teams’ enactments of routines and that result in a lower likelihood of falling prey to the negative side-effects of operating routines. In TTT, there are several individual hands in the game, for which it can be shown that the use of stable operating routines leads to suboptimal performance. Following Cohen and Bacdayan (1994), we set up three ‘traps’ in our experimental setting (hands eight, 15, and 38). These hands can be comparatively easily solved by teams that do not rely on previously established action sequences, such as UU*T action sequences, to place the 2♥ card in the target field and instead choose an alternative approach. To measure attentiveness in action we determined for each team, which percentage of the three ‘traps’ we had set up were successfully avoided. We thus call our measure for attentiveness in action ‘percentage of traps avoided’.

#### 3.2.4 Final questionnaire and remuneration

Upon completion of the TTT game, the participants were asked to fill out a questionnaire, which included manipulation checks and demographical questions. Participants were paid their winnings in cash shortly after the end of the study. Basic data analyzes contributed to determining the amount to be paid. Specifically, we made a fixed payment of €6.15 and paid participants an additional amount according to performance (*M* = €4.02, SD = €1.46, Min = €-3.60, Max = €5.18). To meet the requirements of the laboratory where we collected our data, we guaranteed that each participant would receive at least €6.00. Regarding the incentives, we followed the procedure of Wollersheim and Heimeriks (2016). Specifically, we informed the participants that–in addition to a fixed payment of 6.16 euro–they jointly earn 50 cents with each successfully completed hand. For every move (including passing) they required to successfully complete the hand, 5 cents were subtracted from their payoff. Consequently, participants could maximize their outcome by acting quickly, but still in a thoughtful manner.

### 3.3 Sample

In total, 168 participants arranged into teams of two players participated in the study. Participants were randomly assigned to teams, and the teams were randomly assigned to the experimental conditions. 54 participants (i.e., 27 teams) were assigned to the control condition, 56 participants (i.e., 28 teams) were assigned to the sadness condition, and 56 participants (i.e., 28 teams) were assigned to the fear condition. The participants were recruited using the software ORSEE (Greiner, 2004). For one pair of players, technical problems occurred; they could not finish the experiment and were thus excluded from the dataset prior to the analyzes. The final sample—*N* = 166—consisted of 118 men (71.1%) and 48 women (28.9%), with ages ranging from 18 to 50 and a mean age of 21.91 years (SD = 3.08).

## 4 Experimental results

### 4.1 Summary statistics

Table 2 provides descriptions and correlations for the most important variables. We found correlations among all three dimensions that capture the emergence of operating routines. In contrast, we found no significant correlations between attentiveness in action–the dimension of routine development that captures the modification of operating routines–and the three dimensions that capture the emergence of operating routines. These findings support our assumption that operationalizing the emergence and modification of operating routines separately is reasonable.

**Table 2:** Descriptive statistics and correlations (Level of analysis: team).

		Mean	SD	(1)	(2)	(3)	(4)	(5)	(6)	(7)	(8)
(1)	**Repetitiveness in action** Average no. of repetitions of each action sequence	2.802	0.381	1.000							
									
(2)	**Speed in action** Average move time per hand	5.460	0.739	−0.168	1.000						
				(0.128)							
(3)	**Reliability in action** Average deviation in number of moves relative to best team	1.647	0.650	−0.548	0.213	1.000					
				(0.000)	(0.054)						
(4)	**Attentiveness in action** Percentage of traps avoided	0.388	0.322	−0.168	0.003	−0.074	1.000				
				(0.140)	(0.981)	(0.518)					
(5)	Share of UU*T moves UU*T moves in relation to all moves	0.220	0.052	0.430	−0.094	−0.563	0.026	1.000			
				(0.000)	(0.397)	(0.000)	(0.819)				
(6)	**Money gained** Money gained per team	856.646	156.999	0.527	−0.194	−0.986	0.050	0.572	1.000		
				(0.000)	(0.079)	(0.000)	(0.663)	(0.000)			
(7)	**Sadness** Mean sadness among team members	2.278	1.728	−0.054	0.062	−0.043	0.057	−0.047	0.041	1.000	
				(0.627)	(0.577)	(0.701)	(0.620)	(0.670)	(0.715)		
(8)	**Fear** Mean fear among team members	1.222	1.556	−0.028	0.252	−0.011	0.010	0.218	0.031	0.274	1.000
				(0.805)	(0.022)	(0.922)	(0.933)	(0.048)	(0.781)	(0.012)	

*P*-values in parentheses.

### 4.2 The effects of sadness and fear on routine development

#### 4.2.1 Repetitiveness in action

To understand to what extent routine development differed between experimental conditions, we analyzed the ‘repetitiveness of distinct action sequences’. On average, teams across all conditions repeated each action sequence 2.80 times (SD = 0.38). Teams in the sadness condition (*M* = 2.87, SD = 0.33) repeated their action sequences significantly more often than teams in the control condition (*M =* 2.62, SD = 0.49), *t*(53) = 2.23, *p =* 0.030, *d* = 0.599. We thus observe a medium-sized effect of sadness on repetitiveness in action. We did not find additional significant differences for repetitiveness in action sequences between the other condition comparisons (fear condition: *M =* 2.78, SD = 0.43).

Thus, consistent with our expectations, we observed that teams experiencing sadness generally acted more repetitively than teams in the control condition. Accordingly, teams in the sadness condition developed comparatively more stable operating routines, presumably in order to increase control over their actions. Regarding fear, our findings do not robustly support our expectation that fear would generally lead to more repetitiveness in action.

#### 4.2.2 Speed in action

To test whether sadness and fear affect how automatically operating routines are executed, we followed Cohen and Bacdayan (1994) in analyzing speed in action. Specifically, we observed the ‘average move time per hand’ in the TTT game. For each team and each hand, we measured the average number of seconds the team required to finish each move of that hand. This way, we were able to test absolute speed in action and changes in speed in action throughout the game.

We started our analysis by comparing how teams in our experimental conditions differed regarding their absolute speed in action. Simple group-comparisons revealed that the ‘average move time per hand’ was significantly lower in the sadness condition (*M* = 5.35, SD = 1.87) than in the control condition (*M* = 5.60, SD = 2.25), *t*(2174) = 2.84, *p* = 0.005, *d* = 0.122. We found no significant differences in the ‘average move time per hand’ between the fear condition (*M* = 5.56, SD = 2.13) and the control condition, *t*(2174) = 0.39, *p* = 0.695, *d =* 0.017, but we found significantly quicker moves in the sadness condition relative to the fear condition, *t*(2238) = 2.53, *p* = 0.012, *d* = 0.107. Thus, sadness generally led to comparatively quicker moves, yet the observed effects are small.

To analyze the evolution of the ‘average move time per hand’ throughout the TTT game, we conducted an OLS regression analysis of speed in action, which we present in Table 3. Our regression analysis predicts that across all conditions, with each hand of the game, the ‘average move time per hand’ decreased by 0.10 s (*SE* = 0.00, *p* < 0.001, *R*^2^ = 0.258). Hence, every 10 hands of the game, the ‘average move time per hand’ decreased by roughly 1 sec. With the regression model, we tested for interaction effects between the emotional manipulations and game progress, which in the regression analysis is represented by the variable hand index. We found a significant positive interaction effect between sadness and the hand index (*b* = 0.02, *SE* = 0.01, *p* < *0*.001) and a slightly significant positive interaction effect between fear and the hand index (*b* = 0.01, *SE* = 0.01, *p* < *0*.086). With respect to the overall improvement in “average move time per hand,” the positive coefficients suggest that both, teams in the sadness and in the fear conditions, could not decrease their “average move time per hand” (and thus, increase their speed in action) during the game to the same extent as the teams in the control condition.

**Table 3:** Speed in action: OLS regression analysis of average move time per hand.

	Coeff.	SE
Constant	7.581***	0.108
	(0.000)	
Hand index	−0.103***	0.005
	(0.000)	
Sadness condition (1 = yes vs. 0 = no)	−0.691***	0.151
	(0.000)	
Fear condition (1 = yes vs. 0 = no)	−0.225	0.151
	(0.137)	
Sadness condition × hand index	0.025***	0.007
	(0.000)	
Fear condition × hand index	0.012^+^	0.007
	(0.086)	
Observations	3,296	
*R* ^2^	0.258	

Negative coefficients correspond to more speed in action. *P*-values in parentheses; ^+^ *p* < 0.1, ***p < 0.001.

**Figure 2:**
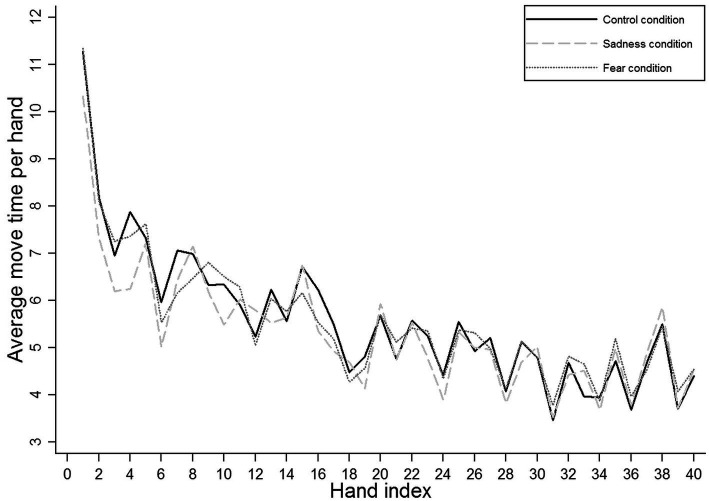
Speed in action: development of the average move time per hand with game progress.

Figure 2 illustrates this finding and provides a deeper insight into the change in “average move time per hand” during different phases of the game. In the first few hands (i.e., in the initial rounds of play), teams in the sadness condition (and to a lesser extent, teams in the fear condition) managed to decrease their “average move time per hand” more than teams in the control condition. However, as the game progressed, teams in the control condition achieved comparable speeds.

Thus, consistent with our expectations, we generally observed more speed in action in the sadness condition relative to the control condition. Contrary to our expectations, teams in the sadness condition not only acted generally quicker than teams in the control condition but also than teams in the fear condition. Yet, at the same time, teams in the sadness condition showed comparatively weaker increases in speed in action with game progress relative to teams in the control condition. Hence, whereas sad teams acted generally quicker than teams in the remaining conditions, this discrepancy in speed emerged at an early stage of the TTT game and tended to decrease over time. Regarding fear, against our expectations, teams in the fear condition acted at speeds comparable to teams in the control condition. At the same time, teams in the fear condition increased their speed in action slightly less strongly over the course of the game than teams in the control condition. Thus, fear did not robustly affect speed in action in absolute terms, but with game progress, it led to a relative decrease in speed in action relative to the control condition.

#### 4.2.3 Reliability in action

To test whether sadness and fear affected how functionally developed operating routines are, we followed Cohen and Bacdayan (1994) in analyzing reliability in action. To measure teams’ reliability in action, we looked at the ‘deviation in number of moves relative to the best team’.

We conducted an OLS regression analysis in which we regressed ‘deviation in number of moves relative to the best team’ on the hand index, on two dummy variables corresponding to our experimental manipulations of sadness and fear, and on terms that test for interactions between our experimental manipulations and the hand index. We present our findings in Table 4. Our regression analysis (Table 4) suggests negative main effects for the sadness (*b* = −1.30, *SE* = 0.21, *p* < *0*.001, *R^2^* = 0.046) and fear manipulations (*b* = −0.70, *SE* = 0.21, *p* = *0*.001) on the ‘deviation in number of moves relative to the best team’, relating to a relative increase in reliability with sadness and fear. To better understand differences in reliability in action between our experimental conditions, we additionally conducted pairwise tests in which we aggregated teams’ mean ‘deviation in number of moves relative to the best team’ over all hands. These tests revealed that teams in the sadness condition required, on average, only 1.46 more moves (SD = 0.37) to finish a hand than the, respectively, best performing team, which is significantly fewer moves than teams in the control condition (*M* = 2.21, SD = 1.64), *t*(53) = 2.37, *p* = 0.021, *d* = 0.634. Regarding the fear condition, the pairwise tests reveal no significant differences in reliability in action between the fear condition (*M* = 1.86, SD = 0.83) and the control condition, *t*(53) = 1.01, *p* = 0.317, *d* = 0.271. Teams in the sadness condition performed significantly more reliably than teams in the control and fear conditions, *t*(54) = 2.33, *p* = 0.024, *d* = 0.623. We thus observed medium positive effects of sadness on reliability in action and no robust effects for fear. Accordingly, teams in the sadness condition generally solved the TTT game in a more reliable fashion than teams in the remaining conditions.

**Table 4:** Reliability in action: OLS regression analysis of deviation in number of moves relative to best team.

	Coeff.	SE
Constant	3.234***	0.148
	(0.000)	
Hand index	−0.058***	0.007
	(0.000)	
Sadness condition (1 = yes vs. 0 = no)	−1.303***	0.207
	(0.000)	
Fear condition (1 = yes vs. 0 = no)	−0.700**	0.207
	(0.001)	
Sadness condition × hand index	0.034***	0.009
	(0.000)	
Fear condition × hand index	0.023*	0.009
	(0.011)	
Observations	3,296	
*R* ^2^	0.046	

Negative coefficients correspond to more reliability in action. *P*-values in parentheses; *p < 0.05, **p < 0.01, ***p < 0.001.

**Figure 3:**
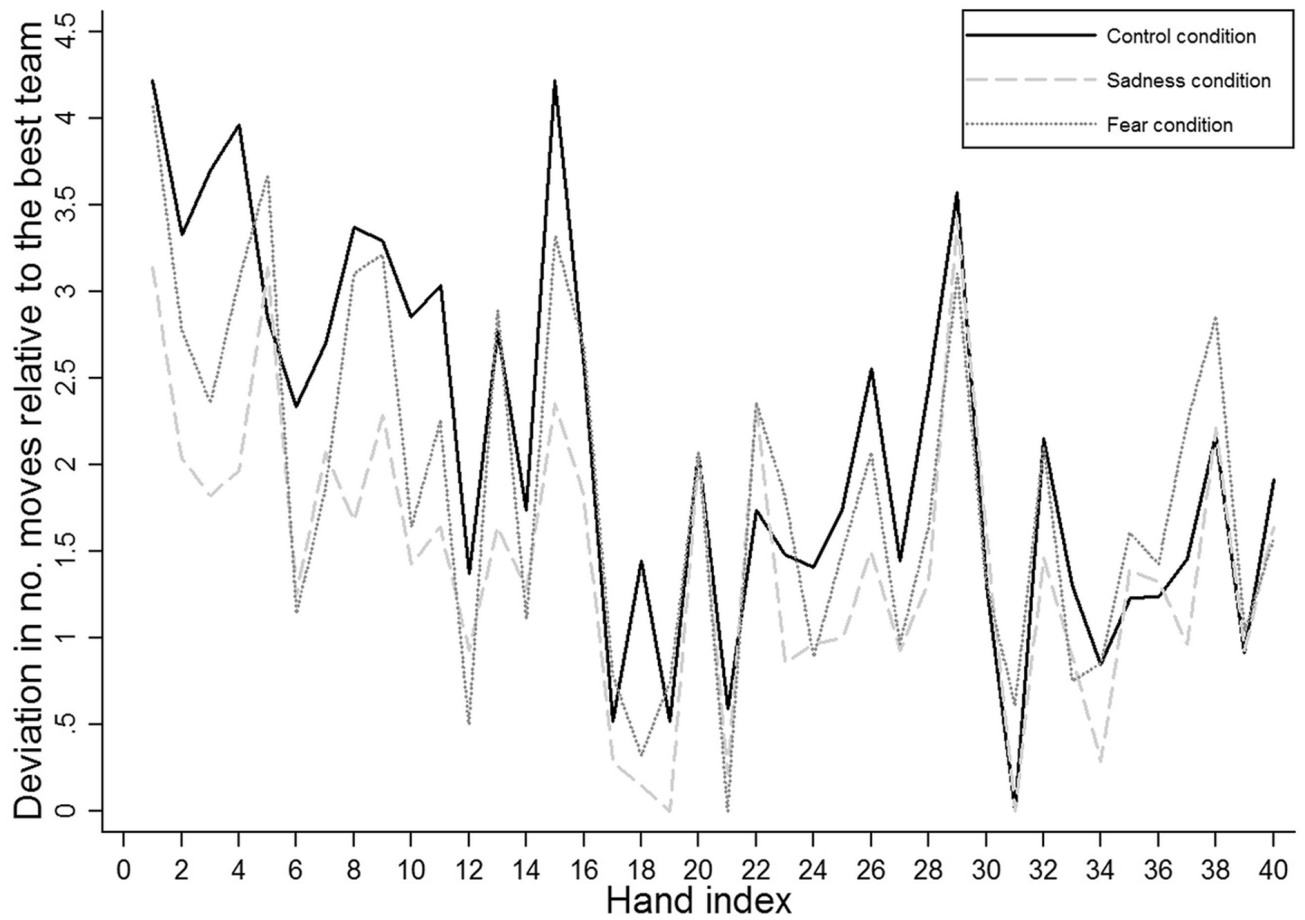
Reliability in action: development of the deviation in number of moves relative to the best team with game progress.

Whereas our regression analysis presented in Table 4 suggests that in all experimental conditions, the ‘deviation in number of moves relative to the best team’ decreased by an average of 0.06 moves (SE = 0.01, *p* < 0.001) with each hand of the game (and consequently, reliability in action increased), the regression yielded positive interaction coefficients for our emotional manipulation sadness and the hand index (*b* = 0.03, *SE* = 0.01, *p* < *0*.001) and for fear and the hand index (*b* = 0.02, SE = 0.01, *p* = *0*.011). These positive coefficients suggest that with game progress (i.e., with increases in the hand index), the ‘deviation in number of moves relative to the best team’ decreased less strongly in our sadness and fear conditions than in our control condition. Hence, we observed two distinct aspects of reliability in action, namely the rate of change in reliability and the relative level of reliability. On the one hand, when considering the entire duration of the game (i.e., rate of change in reliability), we found that over the course of the game, teams in the sadness and fear conditions could not increase their reliability in action to the same extent as teams in the control condition. Figure 3 presents this finding in a more comprehensible way. The graph illustrates how reliability in action in our experimental conditions increased with game progress. Whereas in the sadness and fear conditions the ‘deviation in number of moves relative to the best team’ decreased quickly in the early hands of the game, it took teams in the control condition longer (i.e., more hands) to perform reliably. However, over the course of the game, teams in the control condition showed a steady increase in reliability, and toward the end of the game, they achieved similar levels of reliability.

Thus, on the other hand, against our expectations, teams in the sadness condition developed generally more reliable operating routines than teams in the control and fear conditions, especially during the early stages of the game (i.e., relative level of reliability). These differences in reliability emerged at a very early stage of the TTT game, but they decreased over time. Unexpectedly, teams in the fear condition did not robustly differ from teams in the control condition in terms of their absolute reliability. Yet, with game progress, reliability in action in the fear condition increased comparatively less strongly than in the control condition.

Accordingly, we observed significant differences in the payouts, with teams in the control condition earning less than teams experiencing sadness and fear. It is crucial to acknowledge these differences and to emphasize that those variations are driven by the nuanced effects of speed and reliability in action.

#### 4.2.4 Attentiveness in action

To understand the effects of sadness and fear on the modification of operating routines, we analyzed attentiveness in action. This dimension allows us to address the question of whether sadness and fear affect how effectively operating routines are modified to match the dynamics of their environment. We implemented three ‘trap hands’, i.e., 8, 15, and 38, which can be solved quite easily by teams that act attentively but result in suboptimal performance if teams rely on pre-established operating routines (Cohen and Bacdayan, 1994).

The 79 teams that managed to play all three hands were able to avoid, on average, 38.82 percent (SD = 0.32) of the three traps. We found significant differences in the ‘percentage of traps avoided’ between the sadness and fear conditions, *t*(54) = 2.18, *p* = 0.033, *d* = 0.583. The observed medium-sized effect is quite distinct. The average team in the sadness condition avoided only 29.76 percent (SD = 0.26) of the three ‘traps’, whereas teams in the fear condition avoided 47.62 percent of them (SD = 0.34).

#### 4.2.5 Additional analyzes concerning the patterning dimension of routines

In addition to the analyzes that we conducted to shed light on the performative dimension of routines (i.e., on the actual actions), we performed analyzes with respect to the patterning dimension of routines (i.e., the sequences of action). Routine dynamics research suggests to consider routines as “emergent patterns that form in the actual performance” (Geiger, 2022: 2) and as a process of constant reproduction of actions across temporal and spatial boundaries (Feldman et al., 2016). In this context, we investigated the extent to which participants displayed routinized or flexible behavior. In particular, based on Egidi (1996), we analyzed the two minimal paths for solving the TTT game (i.e., 442 and 422 strategies).^3^

First, we identified Egidi’s (1996) coordination patterns (i.e., 442 and 422) among all teams. To assess whether the teams displayed routinized or flexible behavior, we created a score from the 442 and 422 paths by dividing the number of uses of the 422 strategy by the total number of uses of either 442 or 422 strategies. As a result, we obtained values between 0.59 and 0.96; the closer the value is to 1.0, the more rigid is the routinized behavior of the teams. Second, we calculated the means for the scores in each condition and used t-tests to investigate whether there are any significant differences. We observed that participants in the sadness condition (*M* = 0.71, SD = 0.14) were significantly more flexible in their routines than participants in the control condition (M = 0.77, SD = 0.14, *p* = 0.009). The same is valid for participants in the fear condition (*M* = 0.70, SD = 0.12), who were significantly more flexible in their routines than participants in the control condition (M = 0.77, SD = 0.14, *p* = 0.002). We did not observe significant differences between participants in the sadness condition (M = 0.70, SD = 0.14) and the fear condition (*M* = 0.70, SD = 0.12, *p* = 0.337).

Third, we computed dummy variables for the experimental conditions to calculate a linear regression examining whether routinized behavior as a dependent variable could be explained by the induced emotions, namely sadness and fear. The negative coefficients suggest that teams perceiving sadness (*b* = −0.039, SE = 0.026, *p* = 0.140) and fear (*b* = −0.068, SE = 0.026, *p* = 0.009) tend to have comparatively more balanced paths and, accordingly, more flexible routines. Table 5 gives an overview.

**Table 5:** Coordination patterns: linear regression analysis of the influence of induced emotions on routinized behavior.

	Coeff.	SE
Constant	0.763***	0.019
	(0.000)	
Control condition (dummy variable)	0.069**	0.026
	(0.009)	
Sadness condition (dummy variable)	−0.039	0.026
	(0.140)	
Fear condition (dummy variable)	−0.068**	0.26
	(0.009)	

Negative coefficients correspond to comparatively more balanced paths. *P*-values; ^+^*p* < 0.1, *p < 0.05, **p < 0.01, ***p < 0.001.

To examine the underlying routine performance of participants at an early and advanced stage of the experiment across all conditions, we further conducted descriptive statistics as an additional analysis. According to Cohen and Bacdayan (1994), we repeated the deck of cards from sequences 1–5 in sequences 26–30. Accordingly, the range and mean values indicate that the participants improved across all conditions in terms of sum of move durations divided by the number of moves in the specific hand, money that the group gained for the specific hand, and duration of the specific hand. Tables 6 and 7 provide an overview of the results.

**Table 6:** Comparison of routine performance of participants at early and advanced stages of the experiment.

Games 1–5	Condition	*N*	*Minimum*	*Maximum*	*M*	SD
Sum of move durations divided by the number of moves in the specific hand	Control	135	4.49	23.43	8.55	2.62
	Sadness	140	3.70	13.96	7.76	2.03
	Fear	140	3.89	29.26	8.65	3.09
Money that the group gained for the specific hand (in Cents)	Control	135	−100	30	6.22	31.08
	Sadness	140	−35	30	15.89	12.61
	Fear	140	−100	30	11.32	20.99
Duration of the specific hand (in seconds)	Control	135	19.03	238.41	67.01	43.76
	Sadness	140	16.53	198.51	52.06	29.09
	Fear	140	16.52	175.82	64.53	39.60
Games 26–30	Condition	N	Minimum	Maximum	M	SD
Sum of move durations divided by the number of moves in the specific hand	Control	133	3.46	9.33	5.51	1.31
	Sadness	140	3.20	10.52	5.35	1.20
	Fear	140	3.55	10.18	5.59	1.26
Money that the group gained for the specific hand (in Cents)	Control	133	−75	30	17.33	14.51
	Sadness	140	−5	30	20.21	8.52
	Fear	140	−35	30	19.54	11.01
Duration of the specific hand (in seconds)	Control	133	14.18	125.48	31.59	17.98
	Sadness	140	11.51	81.14	28.52	13.34
	Fear	140	12.02	96.16	29.67	14.24

**Table 7:** Overview of the results.

	Sadness	Fear
Repetitiveness in action	Medium-sized positive effect	No effect
Speed in action	Highly positive effect	Slightly positive effect
Reliability in action	Medium-sized positive effect	Medium-sized positive effect
Attentiveness in action	Medium-sized positive effect	Medium-sized positive effect

## 5 Discussion

**Figure 4:**
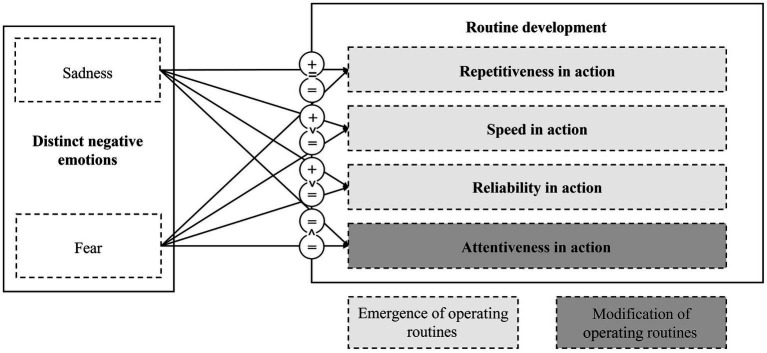
Measured effects of sadness and fear on different dimensions of routine development. The figure illustrates the expected effects of the distinct emotions sadness and fear on different dimensions of routine development. = denotes no effect; + denotes positive effect; − denotes negative effect; > denotes more positive effect when compared to other emotion rather than to control condition.

This study set out to explore the effects of distinct negative emotions on routine development. In the context of the Carnegie perspective, we thus address both the unintended consequences of negative stimuli induced by organizational change and the limitations of human rationality. We focused on sadness and fear due to their different natures–e.g., whereas sadness relates to uncertainty acceptance and risk taking, fear relates to uncertainty and risk avoidance (Raghunathan and Pham, 1999; Lerner and Keltner, 2001)–and due to their high relevance in change processes, in which sadness is related to certain states that are left behind, whereas fear is related to uncertain future states (Verduyn et al., 2009). Figure 4 summarizes the observed differences between our emotional manipulations. Our findings support our underlying assumption that distinct negative emotions differ in their effects on routine development.

Regarding repetitiveness in action, we find that sad teams repeated their operating routines more often than teams in the control condition. A potential explanation for this observation points to situational control (Smith and Ellsworth, 1985). Sadness may generally lead to a perceived shift from human control toward situational control (Smith and Ellsworth, 1985), which in turn may be countered by an increased reliance on repetitive and thus easily controllable actions (Nelson and Winter, 1982; Becker, 2004). Against our expectations, we observe no clear tendency toward more repetitiveness in action in the fear condition. Fear kept teams from developing stable, repetitive routines, potentially due to the fact that fear in our setting led to more conscious, deliberate actions that differed from task to task (*cf.* our findings for attentiveness in action). Our findings suggest that sadness leads to a stronger urge to restore control through repetitiveness in action. In contrast, teams in the fear conditions seem to have tried to restore control by acting more deliberately but less repetitively.

By analyzing speed in action, we explored to what extent sadness and fear affected the ‘off-loading’ of cognitive efforts onto automatized–and hence quickly executed–operating routines (Laureiro-Martinez, 2014). Unexpectedly, sadness also led to quicker actions than fear, whereas we observed no absolute differences in speed in action between the fear and control conditions. However, teams in the fear condition, increased their speed less strongly over the course of the task than teams in the control condition. Thus, teams subjected to sadness were comparatively quicker at cognitively ‘off-loading’ their actions into automatized, quickly executed action sequences. This ‘head start’ in routine development enabled them to act at comparatively higher speeds. However, relative to the control condition, this advantage in speed gradually decreased over time. Given that we observed very similar levels of speed among our experimental conditions toward the end of the experimental task, sadness-induced speed in action seems to have been a temporary phenomenon limited to early stages of routine development. Our finding is consistent with the notion of sadness-induced ‘myopic misery’ (Lerner et al., 2013), which relates to impatience and an increased demand for instant gratification (Lerner et al., 2013). In our setting, this demand was satisfied by quick routine development. With regard to fear, our findings do not suggest any association of fear with temporal myopia (Vuori and Huy, 2016). Thus, fear does not seem to foster routine development by increasing impatience. Moving beyond the results from our laboratory experiment, however, there are numerous internal and external factors to consider contributing to a sufficient contextualization of the phenomenon of temporal myopia. For instance, sadness may contribute to suppression and social isolation (Páez et al., 2013) and consequently slower response time, whereas fear may lead to suboptimal communication resulting in more myopic decisions. From a more general perspective on decision making processes, Opper and Burt (2021) grounded temporal myopia in the context of professional networks and social situations, indicating that managers in closed networks are more likely to be confronted with temporal myopia. Further, the experimental results of Worthy et al. (2012) suggest that increased working memory load tends to cause individuals to focus on the immediate consequences of their actions. Accordingly, previous research results indicate that various factors and their interaction foster the phenomenon of temporal myopia. For instance, it can be presumed that negative emotions, such as sadness or fear, affect working memory and amplify the effect of temporary myopia.

Reliability in action allowed us to test whether sadness and fear affected the functionality of the developed operating routines. Unexpectedly, sadness led to comparatively more-reliable operating routines. However, with game progress, this lead in reliability in the sadness condition became relatively smaller in comparison to the control condition. Against our expectations, fear was not robustly associated with an absolute increase in reliability in action. However, with game progress, we observed a decrease in reliability in action in the fear condition relative to the control condition. This finding is somewhat surprising given that sadness is often associated with uncertainty acceptance and risk taking, in contrast to fear, which is associated with uncertainty avoidance and less risk taking (Raghunathan and Pham, 1999). Accordingly, we would have expected a decreased demand for certain, riskless, and reliable actions with sadness and an increased demand for such actions with fear. If sadness in our setting actually caused a demand for less certainty, as previous literature would suggest, this demand was outweighed by sad teams’ tendency to seek for speed by quickly repeating their predeveloped solutions without much consideration. However, in our setting, this behavior led to reliable outcomes. In contrast, it seems that fearful teams’ demand for more certainty was offset by their tendency to act more attentively, slower, and less repetitively.

Regarding attentiveness in action, we found that sadness did not decrease and fear did not increase attentiveness in action relative to the control condition. However, in support of our expectations, sadness led to less attentiveness in action than fear. Hence, relative to the sadness condition, fear enabled teams to modify their operating routines attentively in order to avoid ‘performance traps’. This finding suggests that the increases in repetitiveness in action, speed in action, and reliability in action that we observed with sadness came at the cost of less attentiveness in action. Apparently, the high degree of routinization associated with sadness led to ‘myopic misery’ (Lerner et al., 2013). Sad teams’ attention was ‘suboptimally’ regulated by dynamic capabilities (Cohen and Bacdayan, 1994), whereas afraid teams, which relied on less-automatized operating routines, were comparatively better able to adjust their operating routines when necessary.

### 5.1 Theoretical implications

First, we show in light of the Carnegie perspective that distinct negative emotions as cognitive stimuli may have distinct effects on different dimensions of routine development, hence providing a better understanding of how emotions affect decision-making and change processes in organizations. With our finding of differential effects of distinct negative emotions, we enhance the growing body of work that demonstrates that operating routines and the dynamic capabilities through which they are regulated entail not only reason but also emotion (Hodgkinson and Healey, 2011, 2014; Døjbak Håkonsson et al., 2016; Parke and Myeong-Gu, 2017). We contribute to this research by showing that distinct negative emotions, such as sadness and fear, vary in their effects on routine development and by showing that these distinct emotions have differential effects on both performative and pattering dimensions of routine development (Egidi, 1996). Whereas sadness promotes the emergence of more repetitive, quicker, and reliable operating routines, fear enables teams to comparatively more attentively modify operating routines. Thus, whereas previous research finds that negative and positive emotions may generally affect the likelihood that teams adopt new routines (Døjbak Håkonsson et al., 2016), our findings suggest that in order to understand how negative emotions affect routines and their development, it is important, first, to differentiate between the distinct negative emotions that accompany routine development, and second, to follow Salvato and Rerup’s (2011) suggestion of separating routines into their individual components and dynamics, which, as we find, may be subject to distinct emotional influences. Hence, our findings advise researchers who are responding to the repeated calls to explore the emotional foundations of organizations (Salvato and Rerup, 2011; Laureiro-Martinez, 2014; Ashkanasy et al., 2017) to not open only one black-box–organizational routines–while keeping emotions, as important antecedents of routines, in another black-box. Instead, our findings encourage researchers to explore the microfoundations of emotions and routines simultaneously in order to reveal their interrelations. Our study is the first to maintain emotion induction over a comparatively long period of time. This approach might be adapted by scholars in psychology and organizational research, as longer-term emotion induction allows for a nuanced examination of emotions and their influence on decision making in organizations, integrating both cognitive dynamics and impulses (Baldessarelli et al., 2022) and providing an important contribution for future research.

Second, we complement previous research emphasizing the importance of emotions in shaping the strategic decisions behind exploration and radical innovation in organizations, i.e., the decisions behind the abandonments of operating routines (Adler and Obstfeld, 2007). We reveal that (distinct negative) emotions may also guide less-radical forms of change in organizations–organizational evolution through routine development. Whereas routine development may generally engender as well as inhibit innovation (Hannan and Freeman, 1984; Feldman, 2000), our findings suggest that distinct negative emotions shift work teams’ actions between stability and flexibility and thus influence whether and how organizations evolve. Whereas sadness leads organizations to ‘off-load’ cognitively demanding strategic decisions onto quickly applied and relatively static ‘production rules’ (Egidi, 1996) that only rigidly adapt to the dynamics of the environment, fear leads to comparatively more attentiveness in the enactment and development of operating routines. Thus, relative to sadness, fear is more likely to result in effective modifications of pre-established operating routines. Both sadness and fear may hence affect strategic decisions between stability and flexibility in organizations. Our results suggest that sadness fosters rather heuristic decision making, whereas fear fosters comparatively more-attentive team-level decision making. Interestingly, these results conflict with other recent research findings in the field of psychology. For example, Treffers et al. (2020) indicated that sadness among managers, especially under high time constraints, leads to improved original strategic decisions, which argues against rigid rule following. Furthermore, Yu et al. (2020) found in their study that fear is associated with lower cognitive flexibility and, as a result, an increased level of impulsivity, which seems to be inconsistent with high attention. Accordingly, our results contribute to the consolidation and contextualization of previous findings. For instance, (negative) emotions might have different effects on decision making among people with and without leadership responsibilities. Moreover, in the context of the Carnegie perspective, we extend previous research findings that suggest that organizational routines are commonly shaped by the management. For instance, the perception of threats (e.g., from changing market conditions) may reinforce routine rigidity. In this context, Gilbert (2005) found that the management centralizes control over decision making, reduces the level of experimentation, and focuses on existing resources when threats are perceived. Complementing these findings, Nigam et al. (2016) found that individuals with role-based authority in particular influence changes in organizational routines. However, our results indicate that negative emotions (induced by organizational changes) are also capable of influencing the development of organizational routines and are not necessarily driven by individuals with high levels of authority. From a more general perspective, we also contribute to the concept of implicit coordination within teams, providing a more nuanced view of dynamically evolving coordination and performance processes. According to Rico et al. (2008), routines within teams may develop in line with the socioemotional behavior of team members. In relation to our study, this may imply that fear and sadness have an impact on implicit, non-verbal interactions and, accordingly, may influence performance processes, apart from explicit working routines and organizational guidelines.

Third, we respond to more-general calls for more research on the (positive) effects of distinct negative emotions (Barsade and Gibson, 2007; Ashkanasy et al., 2017). This experimental study follows several previous studies that stress that negative emotions do not *per se* lead to negative outcomes (Lebel, 2017). We enhance these studies by providing evidence for further, previously unknown, and potentially positive effects of negative emotions. We find that two of the negative emotions that accompany change processes (Fugate et al., 2002), sadness and fear, are not necessarily harmful to routine development–an important component of change processes. Whereas sadness among team members leads to an ‘off-loading’ of cognitively demanding actions onto inattentive operating routines and therefore clears cognitive resources for alternative endeavors, fear enables teams to enact their routines comparatively more attentively (Gable and Harmon-Jones, 2010). Accordingly, we provide a differentiated understanding of how distinct negative emotions may be beneficial and how they may be harmful to organizations.

### 5.2 Practical implications

Our findings suggest that managers should not isolate sad or anxious employees in order to avoid emotional contagion of work teams (Barsade, 2002). Our findings reveal that negative emotions are not negative *per se* and that, in fact, in the right constellation, they may enable teams to better cope with the dynamics of their environment. Openly shared emotions may enable managers to identify the specific aspects of change processes that generate negative emotions and to intervene in order to harness the potentially beneficial effects of negative emotions. Such interventions require an in-depth understanding of the effects of distinct negative emotions. Our findings thus provide managers with a better understanding of when they should intervene (e.g., by inducing positive emotions) and when they should tolerate or even encourage negative emotions (e.g., by inviting organizational members to share their emotions).

For instance, in change processes, in which managers seek the quick development of reliable operating routines, managers might encourage employees to openly share their feelings of sadness; otherwise, they might avoid sadness (e.g., by generating positive experiences). Clearly, negative emotions such as sadness cannot easily be avoided in change contexts, yet managers might nevertheless have an influence on which distinct negative emotions dominate teams’ feelings. For instance, sadness, which is related to the certain past, often follows fear, which is associated with uncertain future states (Verduyn et al., 2009). Accordingly, the timing of negative announcements might determine whether employees are afraid (e.g., of potentially losing a beloved colleague) or sad (e.g., about the certain departure of the colleague). Managers who focus on quickly restoring organizational efficiency might in some situations benefit from substituting fear with sadness, e.g., by creating certainty with regard to a negative event. From a more general attention-based perspective, our study further raises awareness that negative emotions might reduce attentional commitment toward change and, as a consequence, employees’ exploratory behavior (Vuori, 2023). Accordingly, managers need to be vigilant about linking changes in routines to positive emotions that increase both the intensity and quality of attentional engagement.

### 5.3 Limitations and suggested paths for further research

Like all research, this study has some limitations. First, the different effect sizes for sadness vs. fear that we observed in our manipulation check suggest that teams in the sadness condition might have experienced sadness to a greater extent than teams in the fear condition experienced fear. Accordingly, we do not know whether our comparatively weaker manipulations for fear in fact suggest that teams in the fear condition were less emotionalized than teams in the sadness condition. However, our finding of more repetitive, quicker, and more reliable routine development in the sadness condition in contrast to comparatively more attentiveness in action in the fear condition is not consistent with explanations that point to differences in the strength of our emotional manipulations. In fact, our findings become more meaningful when we consider that the emotional manipulations in our experimental setting are likely to be rather weak when compared to emotions that, for instance, are experienced in actual change processes. The levels of sadness and fear that we induced in the laboratory are very likely to be experienced as less intense than the levels of sadness and fear one could expect someone to feel who just lost or is going to lose his or her job. In this context, it is crucial to differentiate between induced emotions in a laboratory setting and the emotions experienced by individuals during real-world organizational change. Our study reveals insights into the effects of induced emotions (that are unrelated to the task) on routine development, whereas we cannot conclude anything concerning the effects of naturally occurring emotions in the context of organizational change. However, as naturally occurring emotions (independent of whether they are task-related or not) are often most presumably stronger than emotions induced in the laboratory. It appears reasonable to assume that our findings are attenuated rather than inflated. Future research may explore the effects of naturally occurring emotions in the context of organizational change to complement our findings, involving longitudinal studies, surveys, or qualitative interviews with individuals undergoing real organizational change. Regarding the data analyzes that we performed, we acknowledge that the substantial differences in payments between the conditions raise an interesting avenue for future research. More specifically, structural equation modeling might help to build and test complex models that capture the relationships between various variables, such as emotional states, action routines, and financial performance. Relying on structural equation modeling, future studies might shed more light on the mechanisms through which emotions influence both cognitive processes and economic outcomes, thereby broadening the scope of our research from routine development to other crucial dependent variables.

Second, one might argue that, in addition to the exogenous manipulation of the emotions that we implemented, endogenously generated emotions might have influenced our results. The reason for this argumentation is that emotions such as anger or sadness might be generated within the game as a result of coordination failures, when participants believe that their partner did not conduct the proper move.^4^ To better understand further emotions that the participants felt during the game, we relied on survey data that we collected from our participants after the completion of the game. With respect to further negative emotions, in particular, we instructed our participants to indicate on a scale ranging from 0 to 10 to what degree they currently feel (1) guilty and (2) angry. Based on *t*-tests, we investigated whether there are significant differences with respect to these emotions among the teams that were among the lowest-performing 25% in terms of money gained. Concerning both, anger and guilt, we did not observe any significant differences between the experimental conditions. Thus, we feel confident that endogenously generated emotions did not influence our findings.

Third and finally, some findings of this study are bound by the methodological design and specifically by our experimental task. Specifically, our experimental setting might be limited in its explanatory power, as it isolates teams from the ‘messiness’ that typically characterizes work life. In our experimental setting, we replicated Cohen and Bacdayan’s (1994) experimental setting, which did not feature any obvious form of authority and which prohibited participants from talking during the experimental session. Here, we decided to observe routine development isolated from direct authority and open communication to highlight a characteristic of routines that is often overlooked in empirical studies–the routine as an “organizational unconscious,’ a body of largely inarticulate know-how that underpins so much of an organization’s capabilities” (Cohen and Bacdayan, 1994, p. 566). In this context, the incentive scheme is relevant as well. For our study, adding a variable payment to the basic payment was reasonable, because the variable payment motivated participants to play quickly and carefully at the same time, which favors the development of routines. However, one should consider that other incentive schemes could have changed the results. For example, abandoning the variable payment and thus, relying on a fixed payment solely, would probably have had a negative impact on routine development. Similarly, paying a higher proportion of the money gained to the participant who placed the final card in the target area could have changed the results, because an unequal distribution of the money gained would have led to increased competition between the participants, influencing the action sequences and, accordingly, the dimensions of routine emergence and adaptation. Thus, for the purposes of our study, we feel confident that we relied on an established and suitable incentive scheme. However, future research might analyze the effect of changing the incentive scheme to address similar research questions. In this context, introducing competition–and thus creating a coopetition environment–to the game appears to be particularly promising. Our setting illustrates that in an experimental environment, teams may both develop stable operating routines and coordinate on modifications of these routines in situations where they would lead to undesirable performance. This coordination is enabled by implicit authority and hidden communication. Future research should nevertheless shed more light to the interplay of emotions, open communication and direct authority in routine development processes. Furthermore, relevant boundary conditions occur in relation to task duration and organizational routines: First, in terms of time sensitivity, in short-term tasks with tight timelines (as in our experimental design), teams have less time to adapt and change their routines in the long term in response to emotional experiences. Second, the low complexity of the card game may be more vulnerable to immediate effects of emotion, whereas complex, interdependent routines in organizations may require more time to adapt and may reveal emotional effects over a longer period of time. Third, task familiarity may influence emotional responses, as in well-established and familiar routines emotions have a different, potentially weaker impact. Fourth, an organization’s culture may influence how emotions are handled. Organizations with a strong culture of adaptability and emotional intelligence may exhibit different patterns of routine development in response to emotions than organizations with a rigid or resistant culture. Fifth, it is crucial to acknowledge that the outcomes of emotions in the workplace can be complex and multifaceted. While our study sheds light on specific aspects of how negative emotions can influence routine development, it does not encompass the entirety of potential workplace outcomes. Negative emotions may have diverse effects on various monetary and non-monetary performance variables that extend beyond the scope of our investigation. For instance, creativity and innovativeness, team cohesion, and trust among employees are integral components of workplace performance and we do not know how fear or sadness influence these variables. Future research should consider these aspects in the context of field research or adapted experimental designs.

## 6 Conclusion

The present study represents an important step toward an understanding of the causal influence of sadness and fear on routine development, which represents a crucial mechanism behind organizational change processes. Using a laboratory experiment in which we induced distinct negative emotions in teams, we find that sadness and fear have distinct effects on routine development. Whereas sadness in teams leads to the development of comparatively more repetitive, quicker, and more reliable operating routines, fear enables teams to better recognize and react to ‘performance traps’, i.e., situations in which pre-established operating routines are ineffective. Thus, the study contributes to an increased understanding of how negative stimuli influence individual behavioral responses and subsequent heuristic decision-making. Furthermore, our findings enable researchers and practitioners to better understand and predict the effects of sadness and fear in change processes and contribute toward new theories and practices that will enable organizations to better harness the emotional capacities of their members (Hodgkinson and Healey, 2014).

## Data availability statement

The original contributions presented in the study are included in the article, further inquiries can be directed to the corresponding author.

## Author contributions

JS-W developed the research question and the research design in collaboration with IW and MS. JS-W provided the experimental software used for this essay and conducted the lab experiment. PO and JS-W performed the statistical analyses and wrote the first draft of the manuscript. MR wrote sections of the manuscript. All authors contributed to manuscript revision, read, and approved the submitted version.

## Footnotes

^1^Please note that Wollersheim and Heimeriks (2016) rely on Cohen and Bacdayan (1994) to capture the regulation of routines through dynamic capabilities. In contrast to this study, they use an experimental design that challenged participants’ capacity to identify appropriate procedures following a change in the environment and that required modification of operating routines. In their experiment, they primarily base their identification of the characteristic qualities of dynamic capabilities on Cohen and Bacdayan’s (1994) dimensions by analyzing differences between groups in low vs. high dynamic capability conditions in this change situation. In this study, we use a comparatively stable environment in which participants jointly form and modify routines. In this comparatively stable environment, we employ three of Cohen and Bacdayan’s (1994) dimensions, (1) repetitiveness in action, (2) speed in action, and (3) reliability in action, to describe the emergence of operating routines, given that these dimensions describe aspects of operating routines that may be observed irrespective of change. (4) Attentiveness in action, in turn, describes to what extent teams are able to *deliberately* adjust routines in the more stable, yet due to the different card configurations changing environment. Thus, in this study, we relate this dimension to the modification of routines.

^2^Please note that, additionally, at the end of the experiment, we instructed the participants to inform us about what they believe the objective of the study was. Based on the data that we gathered in this way, we feel confident that nobody understood that we sought to manipulate emotions.

^3^In the TTT experiment, two distinct strategies, known as “442” and “422,” can be employed to accomplish the objective (Egidi and Narduzzo, 1997). Following the 442 strategy means that the Numberkeeper initiates solving the card game. Specifically, the Numberkeeper searches for and places a specific card (e.g., 4♥) into the target position. Subsequently, the Colorkeeper follows suit by searching for and placing a different card (e.g., 2♥) into the target position. This sequence of actions results in a specific card sequence for the target position (e.g., 4♣-4♥-2♥), which explains the name of the relative strategy (i.e., 442). Accordingly, 422 strategy means that the Colorkeeper takes the lead by identifying and placing a specific card (e.g., 2♣) into the target position. Subsequently, the Numberkeeper takes their turn, searching for and placing a distinct card (e.g., 2♥) into the target position. Thus, the card sequence on the target position is, for example, 4♣-2♣-2♥ (i.e., 422 strategy).

^4^We thank an anonymous reviewer for this valuable comment.
